# Global report on preterm birth and stillbirth (1 of 7): definitions, description of the burden and opportunities to improve data

**DOI:** 10.1186/1471-2393-10-S1-S1

**Published:** 2010-02-23

**Authors:** Joy E Lawn, Michael G Gravett, Toni M Nunes, Craig E Rubens, Cynthia Stanton, the GAPPS Review Group

**Affiliations:** 1 Saving Newborn Lives/Save the Children, 11 South Way, Pinelands Cape Town, South Africa; 2 Department of Obstetrics and Gynecology, University of Washington, Seattle, Washington USA; 3 Global Alliance to Prevent Prematurity and Stillbirth, an initiative of Seattle Children's, Seattle, Washington, USA; 4 Department of Pediatrics at University of Washington School of Medicine, Seattle, Washington, USA; 5 Department of Population, Family and Reproductive Health, The Johns Hopkins Bloomberg School of Public Health, Baltimore, Maryland, USA

## Abstract

**Introduction:**

This is the first of seven articles from a preterm birth and stillbirth report. Presented here is an overview of the burden, an assessment of the quality of current estimates, review of trends, and recommendations to improve data.

**Preterm birth:**

Few countries have reliable national preterm birth prevalence data. Globally, an estimated 13 million babies are born before 37 completed weeks of gestation annually. Rates are generally highest in low- and middle-income countries, and increasing in some middle- and high-income countries, particularly the Americas. Preterm birth is the leading direct cause of neonatal death (27%); more than one million preterm newborns die annually. Preterm birth is also the dominant risk factor for neonatal mortality, particularly for deaths due to infections. Long-term impairment is an increasing issue.

**Stillbirth:**

Stillbirths are currently not included in Millennium Development Goal tracking and remain invisible in global policies. For international comparisons, stillbirths include late fetal deaths weighing more than 1000g or occurring after 28 weeks gestation. Only about 2% of all stillbirths are counted through vital registration and global estimates are based on household surveys or modelling. Two global estimation exercises reached a similar estimate of around three million annually; 99% occur in low- and middle-income countries. One million stillbirths occur during birth. Global stillbirth cause-of-death estimates are impeded by multiple, complex classification systems.

**Recommendations to improve data:**

(1) increase the capture and quality of pregnancy outcome data through household surveys, the main data source for countries with 75% of the global burden; (2) increase compliance with standard definitions of gestational age and stillbirth in routine data collection systems; (3) strengthen existing data collection mechanisms—especially vital registration and facility data—by instituting a standard death certificate for stillbirth and neonatal death linked to revised International Classification of Diseases coding; (4) validate a simple, standardized classification system for stillbirth cause-of-death; and (5) improve systems and tools to capture acute morbidity and long-term impairment outcomes following preterm birth.

**Conclusion:**

Lack of adequate data hampers visibility, effective policies, and research. Immediate opportunities exist to improve data tracking and reduce the burden of preterm birth and stillbirth.

## Why focus on preterm birth and stillbirth?

**Figure 1:**
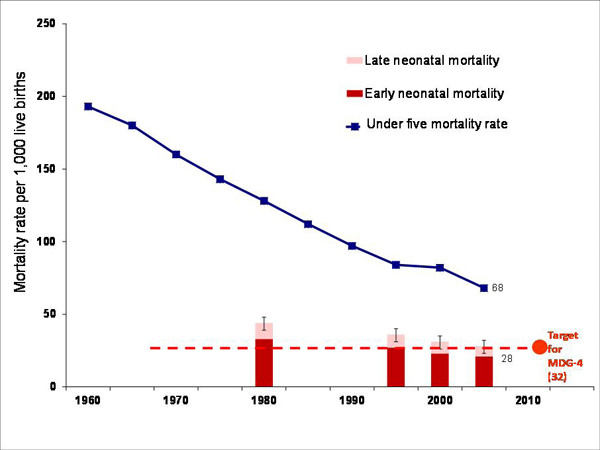
**Early and late neonatal mortality rates and under 5 mortality rates per 1000, 1960-2007.** Source: Lawn, Kerber et al. [1]; Data from UN databases updated to 2007.

While under-5 mortality rates are improving in many countries worldwide, neonatal mortality rates (deaths in the first 28 days of life) have shown much less progress [1]. Neonatal deaths now account for more than 42% of under five deaths (Figure 1), up from 37% in the year 2000 when the Millennium Development Goals (MDGs) were set [2,3]. MDG 4 targets a two-thirds reduction of under-five deaths between 1990 and 2015.

Complications of preterm birth are the leading direct cause of neonatal mortality, accounting for an estimated 27% of the almost four million neonatal deaths every year, and act as a risk factor for many neonatal deaths due to other causes, particularly infections [4]. Hence, achievement of MDG 4 is strongly influenced by progress in reducing neonatal deaths; and since preterm birth is the leading cause of these deaths, progress is dependent on achieving high coverage of evidence-based interventions to prevent preterm delivery and to improve survival for preterm newborns [5]. In some high-income countries (HICs), preterm birth has been high on the maternal, newborn and child health (MNCH) agenda for two decades, but is now starting to receive wider public health attention because of increasing preterm birth rates, particularly in the United States [6]. However, only recently has this issue started to reach the attention of higher-level policy makers in low- and middle-income countries (LMICs). Many countries, particularly in Latin America, have recognized the importance of preterm birth and are looking for solutions in prevention as well as improved care. Understanding and improving the current data are critical to setting priorities for action and for tracking progress.

Another adverse pregnancy outcome that is closely linked to preterm birth is stillbirth, which remains invisible on global policy agendas, as stillbirths are not included in MDG targets or tracking [7]. Each year there are an estimated 3.2 million stillbirths—almost as many as neonatal deaths. Attention to stillbirths has increased notably in the last few years. Important signs of change include the fact that estimation of disability-adjusted life years for stillbirth were calculated and included in the most recent edition of Disease Control Priorities for Developing Countries [8].

It is widely recognized that MDG 5 to improve maternal health has shown the least progress among all MDGs [9]. Maternal mortality is strongly correlated with stillbirth [10]. Increasing attention for preterm birth and stillbirth interventions, alongside increasing investment for mothers, will accelerate progress for these inextricable maternal, fetal, newborn and child health outcomes. Improved data on these pregnancy outcomes are crucial to guiding investment and tracking progress.

This is the first of seven articles in a global report on preterm birth and stillbirth. In this article we present estimates of the current burden, assess the quality of these estimates, review trends, and make recommendations to improve data. The second article discusses the process of pregnancy and childbirth, etiologies of preterm birth and stillbirth and opportunities through discovery science to identify pathways, and potential interventions [11]. Other articles discuss effectiveness of existing interventions [12], barriers and opportunities for scaling up interventions [13], advocacy [14], and ethical considerations [15]. The final article presents a Global Action Agenda created by about 200 MNCH stakeholders [16].

## Preterm birth and stillbirth: assessing the status and quality of global estimates

Less than 5% of the world's births occur in countries with complete vital registration or networks of representative, facility-based data. One-third of the world's births occur at home. Therefore, global-level data rely heavily on household surveys and modelled estimates. Global epidemiological estimation is a new science and builds on principles established for reviewing evidence for public health interventions—particularly with its focus on systematic literature reviews. However, approaches to standardizing the steps and assessing the quality of estimates are yet to be well-defined [17].

GRADE is a system designed to review the quality of evidence supporting health interventions [18]. Here, we have adapted the GRADE system to provide a summary assessment of the quality of major epidemiological parameters related to preterm birth and stillbirth, including rates, causes and impairment outcomes. The following two sub-sections, Preterm Birth and Stillbirth, start with a summary "Epidemiological GRADE" table (Table 1 and Table 5, respectively). They assess the input data and methods used to generate current global estimates for these parameters, current gaps and new work in progress.

## Preterm birth burden

### Defining preterm birth

The preterm birth rate is defined as the percentage of babies born before 37 completed weeks of gestation (Table 1). In addition, more granularity would be helpful for programs, such as dividing moderately preterm (33 to 36 completed weeks of gestation), very preterm (<32 weeks) and extremely preterm (<28 weeks). Particularly in countries where caesarean section is common, differentiating spontaneous and medically induced preterm birth is of programmatic relevance. Trend analysis in Canada suggests that a significant contribution to increasing preterm birth prevalence is related to more aggressive policies for caesarean section for poor fetal growth— which may reduce stillbirth but increase preterm birth [19,20]. Although there is consensus on the broad definition, it is clear that preterm birth is a manifestation of a complex network of causal pathways. Consensus around the phenotypes and comparable case definitions are an important next step in better understanding this syndrome of preterm birth [11].

**Table 1:** GAPPS quality assessment of epidemiological parameters in global estimates using adapted version of GRADE: PRETERM BIRTH

	Prevalence	Preterm birth as a direct/indirect cause-of-death				Impairment following preterm birth	
			
Epidemiological Parameters	Preterm Birth Prevalence Rate	Preterm Birth as Direct Cause of Neonatal Death 2000	Preterm Birth as Direct Cause of Neonatal Death 2005	Preterm Birth as Risk Factor for Neonatal Death	Retinopathy of Prematurity	Chronic Lung Disease	Multi-Domain Impairment
Definition	The proportion of babies born at less than 37 weeks of completed gestation, per 100 live births	Preterm birth direct co006Dplications as the cause-of-death as defined in ICD ie RDS/HMD, necrotizing enterocolitis, intraventricular hemorrhage and other direct complications of preterm birth, or very early neonatal death in newborn of gestational age <32 weeks		Neonatal death in a preterm baby where preterm birth is indirect - for example a baby who is moderately preterm and dies of infection a few days after birth.	A vasoproliferative disorder due to aberrant vascular proliferation of the immature retina. There are 5 stages of severity. The risk of ROP is increased for preterm babies exposed to hyper-oxygenation.	CLD is defined as persistent need for oxygen therapy in order to maintain oxygen saturation above 88% to 36 weeks of postmenstrual age (note some variation in case definitions, also sometimes called Bronchopulonary dysplasia)	Impairment aff ecting more than one domain of function as used in GBD assessments (cognitive, motor, vision, hearing, seizure disorder)
Systematic global estimates available (source and date)	WHO and Child Health Epidemiology Reference Group (CHERG) for Global Burden of Disease (GBD), in process	CHERG for WHO/UN [4]	CHERG and WHO in process	CHERG new grant - not yet started	CHERG for GBD, in process	CHERG for GBD, in process (review by Maneesh Batra)	CHERG for GBD, in process
Countries with VR data used (used as reported, new analysis or adjusted)	Not in vital registration	45 (~96,797 neonatal deaths)	61 (~142,000 neonatal deaths)	Work to be done 2009 - 2012	Not reported consistently from ICD data although ICD codes allocated	Several ICD codes allocated. Not reported consistently from ICD data	Not in vital registration
Countries with survey data used (used as is, new analysis or adjusted)	Not in current national surveys	Not in current national surveys	Not in current national surveys	Work to be done 2009 - 2014	Not in current national surveys	Not in current national surveys	Not in current national surveys
Countries where modelled estimates used (basis of model)	(in process)	138 (27 from VR model and 111 from study based model) Multinomial model to simultaneously estimate 7 causes.	132 (32 from VR model and 100 from study-based model). Multinomial model to simultaneously estimate 7 causes.	Work to be done 2009 - 2014	(in process)	(in process)	(in process)
Types of data inputs for modelling	(in process)	VR, published studies from community (verbal autopsy) and facilities, unpublished datasets (eg DSS)	VR, published studies from community (verbal autopsy) and facilities, unpublished datasets (eg DSS)	Work to be done 2009 - 2014	Often hospital registries	Very few comparable studies even in HICs	Varied and no population based studies cohort from LICs identified
No of studies/ datasets included in global estimate modelling	(in process)	Searches of 6820 hits, 46 studies and 10 unpublished datasets included	Searches of 5591 hits, 71 studies/datasets included	Work to be done 2009 - 2014	(in process)	(in process)	(in process)
Total with measure of parameter (e.g., SBs, preterm births)	(in process)	VR (~96,797 neonatal deaths) Study data (13,685 neonatal deaths)	VR (~130,000 neonatal deaths) Study data (23,638 neonatal deaths)	Work to be done 2009 - 2014	(in process)	(in process)	(in process)
Median yr of input data	(in process)	VR median year 1999 Study data median year 1991	VR median year 2004 Study data median year 1991	Work to be done 2009 - 2014	(in process)	(in process)	(in process)
Variability in outcome measurement methods	(in process)	Yes – death certificates/ ICD codes, clinical assess, med records, verbal autopsy	Yes - death certificates/ ICD codes, clinical assess, med records, verbal autopsy	Work to be done 2009 - 2014	(in process)	varied case definitions	Very varied case definitions, varied age at assessment and multiple assessment tools
Limitations re population representativeness	(in process)	VR data representative for 46 countries. Study based data often from non representative populations	VR data representative for 69 countries. Study based data often from non- representative populations	Work to be done 2009 - 2014	Hospital studies and in LIC/MIC from referral centres, few data re moderate gestational age/BWT babies	Hospital studies and only in high income countries	LIC/MIC from referral centers and limited cohort data
Generalizability to Population of Interest (i.e., geographic match of data to burden)	(in process)	Important gaps in the input data—especially in China, west and central Africa and central Asia	Much improved data for China and India. Still limited for central Africa and central Asia	Work to be done 2009 - 2014	Limited	Very limited	Very limited
Is there systematic equity assessment	No	No	No	No	No	No	No
Global estimate	(in process)	1.12 million (27.9% of 4 million)	1.23 million (33.1% of 3.72 million)	Work to be done 2009 - 2014	(in process)	(in process)	(in process)
Range	(in process)	0.74 to 1.38 million	0.84 to 1.52 million	Work to be done 2009 - 2014	(in process)	(in process)	(in process)
Consistency between estimates if more than one set	To compare to WHO global and regional estimates 2009	First set of systematic estimates	In comparison with 2000 estimates, a reduction in neonatal tetanus deaths, and apparent increase in deaths due to preterm birth	None to compare with	None to compare with	None to compare with	None to compare with
Overall summary of quality of data input	(in process)	Moderate for high income countries, low for low income countries	Moderate for high income countries, low for low income countries	(in process)	(in process)	(in process)	(in process)
Overall quality of estimates according to standards for global estimates	(in process)	Moderate to high - transparent methods, advances in multi-cause modelling now the std for multi-cause work but limited by input data and especially consistency in cause-of-death attribution	(in process)	(in process)	(in process)	(in process)	
Priority areas to improve measurement now	1. Increase quality and quality of gestational age data in routine data sources 2. Test feasibility/ validity of gestational age data collection through household surveys 3. Test feasibility/ validity of simplified gestational age clinical assessment 4. Increase dissemination of country level best estimates once completed	1. Disseminate case definition and hierarchies for preterm complications as a direct cause-of-death vs preterm birth as a risk factor, 2. Increase quality and quality of cause-of-death data in vital registration and national audit data 3. Increase quality and quality of SBR data in verbal autopsy data applying and a standard hierarchy 4. Increase dissemination of country level best estimates		Same inputs as required to improve measurement of gestational age and causes of death	1. Agree on case definitions and measurement tools and ages at which measurement should occur 2. Increase quality and quality of impairment data especially in transitional countries with increasing neonatal survival 3. Disseminate case definitions well so that future studies result in more comparable data, and ideally standard assessment guidelines 4. Advocate for more funding for cohort studies tracking impairment outcomes from preterm birth and other neonatal morbidities		

### Preterm birth prevalence rates

A recent publication estimates about 13 million preterm babies are born each year worldwide [21]. However, country-level data are unavailable for most LMICs. Globally, around one-third of babies are born at home with little or no information on birthweight, gestational age or even survival. For those born in health care facilities, data on birthweight are often lacking or not recorded and compiled. Gestational age is rarely recorded and where recorded, tends to be based on self-reported last menstrual period (LMP), which is fairly imprecise. Differing methods such as LMP, clinical assessment, and ultrasound assessment have varying levels of accuracy. Despite the data gaps, estimates of the prevalence of low birth weight (LBW) are published each year in UNICEF's State of the World's Children report for most nations [22]. These estimates rely on available data in national household surveys, especially the Demographic and Health Survey (DHS) and UNICEF's Multiple Indicator Cluster Survey, applying adjustments for maternal reporting of the child's size and for heaping of birth weights on multiples of 500 grams [23]. Birth weight is only an indirect surrogate for gestational age, and many neonates—those either small or large for gestational age—will be incorrectly misclassified as preterm or term, respectively.

Preterm birth rates in the published literature range from 5% in HICs to 25% in LMICs [24,25]. Population-based data for most LMICs are scarce, especially from Africa. The current status of the global data is summarized in Table 1. The lack of systematic country estimates for the prevalence of preterm birth is an important gap in the visibility of preterm birth. The WHO Special Programme of Research, Development and Research Training in Human Reproduction has recently published estimates of preterm prevalence at global and regional levels (Table 2) [21]. Rates are highest in least developed regions, especially Africa, but are also high in North America. A new exercise in partnership with the neonatal team at the Child Health Epidemiology Reference Group (CHERG) is a systematic review and modelling of preterm prevalence for WHO country-level estimates and that will also be used in the Global Burden of Disease (Table 1).

**Table 2:** Regional variation in the estimated preterm birth prevalence rates

Region	Preterm births (x1000)	Preterm birth rate (%)	95% Confidence Intervals
World Total	12,870	9.6	9.1 - 10.1
More developed regions	1,014	7.5	7.3 - 7.8
Less developed regions	7,685	8.8	8.1 - 9.4
Least developed regions	4,171	12.5	11.7 - 13.3
Africa	4,047	11.9	11.1 - 12.6
Asia	6,907	9.1	8.3 - 9.8
Europe	466	6.2	5.8 - 6.7
Latin America & the Caribbean	933	8.1	7.5 - 8.8
North America	480	10.6	10.5 - 10.6
Oceania (Australia/New Zealand)	20	6.4	6.3 - 6.6

Source: Beck S. et al. [21]

### Preterm birth rate disparities within countries

Preterm birth rates vary greatly within countries and by sociodemographic characteristics. For example, in the United States, great disparities exist between racial and ethnic groups—in both preterm birth rates and outcomes. The most striking differences are between African American women and non-Hispanic white, Asian and Pacific Islander women. In 2005, the preterm birth rates among these groups varied from 18.4% among African American to 11.7% among non-Hispanic white women and 10.8% among Asian and Pacific Islander women [26]. The overall preterm birth rate has increased since 1990, due primarily to a 38% increase in non-Hispanic white preterm births and a 10% increase in Hispanic preterm births [26].

American Indians and Alaska Natives also have high preterm birth rates, reported to be 13.5% in 2005 [27]. Among US Indigenous populations, Native Hawaiians experience the highest infant and neonatal mortality rates [27,28]. The Pregnancy Risk Assessment Monitoring System (PRAMS) conducted by the US CDC estimates that one-half of infant deaths among Indigenous populations in the United States are attributable to low birth weight or preterm birth.

The recording of births and deaths, as well as the likelihood of medical intervention have been shown to be affected by medical caregivers' perceptions of viability of the baby. Babies that are very preterm may be less likely to be recorded or even to receive care despite reasonable chances of survival [29,30]. In countries without neonatal intensive care, few babies below the gestational age of 32 weeks survive and even at 30 weeks may be called "abortions" and not recorded [31]. This is very different than countries with intensive care, where although few babies born alive at 22 weeks may survive intact, by 25 weeks the majority survive [32,33]. Hence even extremely preterm babies may be aggressively resuscitated and data fully recorded, although practices still vary between countries. The Nuffield Council on Bioethics recommends that below 22 weeks of gestation resuscitation should not be attempted, even if a baby is born with signs of life [34].

### Preterm birth prevalence trends

Table 3 provides trends in preterm birth for a number of selected HICs and LMICs, including preterm prevalence in non-representative populations published by WHO in 1995 [35]. Reported preterm birth rates among European and other HICs range from 5% to 9%, and similar to the United States, have been on the rise over the past three decades [36]. A significant contribution to the rise in preterm birth rates reflects an increase in preterm delivery due to medical indication of either the mother or the fetus. In absolute terms, however, medically-indicated preterm births made up less than half of all preterm births in the year 2000 in the United States [36,37].

**Table 3:** Trends in preterm births for selected countries

	Preterm Births (*Percent*)		
	
Country	Previously Reported Rates	Recently Reported Rates	Proportionate Change from Previous Rate
**High-Income Countries**			
Australia [79]	5.9 (1994)	6.6 (2003)	11.8%
Canada [19]	6.3 (1982-1983)	6.8 (1992-1994)	7.9%
Finland [80]	9.1 (1966)	5.2 (2001-2005)	-42.8%
France [81]	7.9(1972)	4.0 (1988-1989)	-49.4%
Israel [82]	11.5 (1986-1987)	9.4 (2003-2004)	-18.3%
Japan [83]	4.1 (1980)	5.4 (2000)	24.4%
New Zealand [84]	4.3 (1980)	5.9 (1994)	37.2%
Scotland [85]	4.9 (1980-1984)	5.6 (2000-2003)	14.3%
United Kingdom	4.6 (1971-1976) [35]	6.0 (2002) [86]	30.4%
United States [87]	(1990)	(2005)	
Non-Hispanic white	8.5	11.7	37.6%
Non-Hispanic black (African American)	18.9	18.4	2.6%
Hispanic	11.0	12.1	10.0%
All races	10.6	12.7	19.8%
Sweden [88]	6.3 (1984)	5.6 (2001)	-11.1%
**Middle-Income Countries**			
Brazil, Pelotas [89]	11.4 (1993)	14.7 (2004)	26.9%
Brazil, Ribeirão Preto [90]	8.0 (1978)	14.8 (1994)	85.0%
Brazil, regression based on all studies [38]	4.0 (1980s)	12.0 (2000s)	200.0%
Chile [91]	5.6 (1990)	6.0 (2000)	7.1%
China	7.5 (1981-1982) [35]	3.5 (1998) [92]	-53.3%
Indonesia	18.5 (1983) [35]	14.2 (1995) [93]	-23.2%
Uruguay (unpublished data)	10.1 (1986-93)	10.3 (2000-2003)	2.0%
Latin America database [39]	9.4 (1985-1990)	9.5 (1996-2003)	1.1%
**Low-Income Countries**			
Bangladesh	22.0 (1994-1997 [94]	16.5 (2000) [95]	-33.3%
Gambia	13.5 (1976-1984) [35]	12.3 (1976-2003) [96]	0.91%
Nepal (rural)	15.8 (1990)- rural 21.8 (1990)-urban [35]	23.1 (1998-2001) [95,97]	-8.9%
Pakistan	10.2 ([98]1992-94)	15.7 (2001-02) [99]	53.9%

In LMICs, data on trends in preterm birth are very limited and results are mixed. In general, LMIC rates tend to be higher than in HICs. In Latin America, rates are increasing in Brazil, possibly related to elective cesarean sections and labor inductions [38]. In an analysis of more than 1.7 million births that took place in 51 maternity hospitals in Latin America, for which Uruguay and Argentina contributed half the births, the rates of preterm birth were essentially the same between 1985 and 2003 (around 9%). However, there was a marked increase in the proportion of preterm births associated with induction/elective caesarean sections during this period [39]. For countries outside of Latin America, such as China, Indonesia, and Bangladesh, the available studies use sub-national samples and should be interpreted with care.

### Preterm birth as a cause-of-death, acute morbidity, and disability

**Figure 2:**
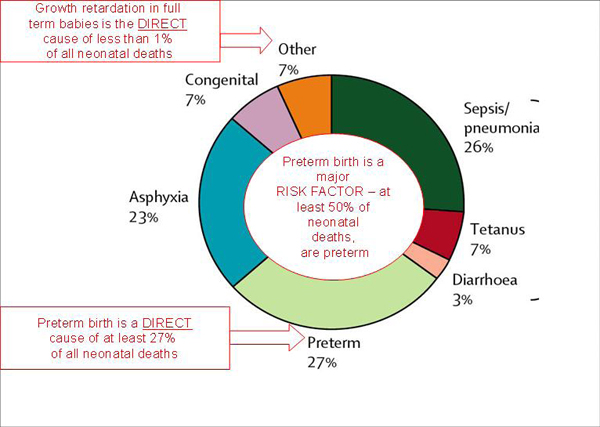
**Causes of neonatal death globally based on estimates for 193 countries around the year 2000.** Source: Reprinted from The Lancet, 365, Lawn JE, Cousens S, Zupan J, 4 million neonatal deaths: When? Where? Why?, 10, 2005, with permission from Elsevier. [2].

Systematic estimates for the causes of neonatal deaths in 192 countries were undertaken by the CHERG based on vital registration data for 45 countries (N=96,797 deaths) and modelled estimates for 146 countries (input database of N=13,685 deaths). These were published in *The Lancet Neonatal Survival Series *[2], incorporated in the *World Health Report 2005 *[40], and in *Disease Control Priorities in Developing Countries *[8,41] (Figure 2). The methods are described in detail elsewhere and also summarized in Table 1. At the global level, these estimates place preterm birth as the single largest direct cause of the world's four million neonatal deaths [2].

In addition to being the leading direct cause of neonatal deaths (Figure 2), preterm birth also increases the risk of dying due to other causes, especially from neonatal infections [2]. An example is a moderately preterm baby who dies of infection after a few days of life. Hence, as well as being the leading direct cause of neonatal deaths, preterm birth is a crucial risk factor for neonatal deaths due to infection. A systematic risk factor analysis is planned (Table 1).

**Figure 3:**
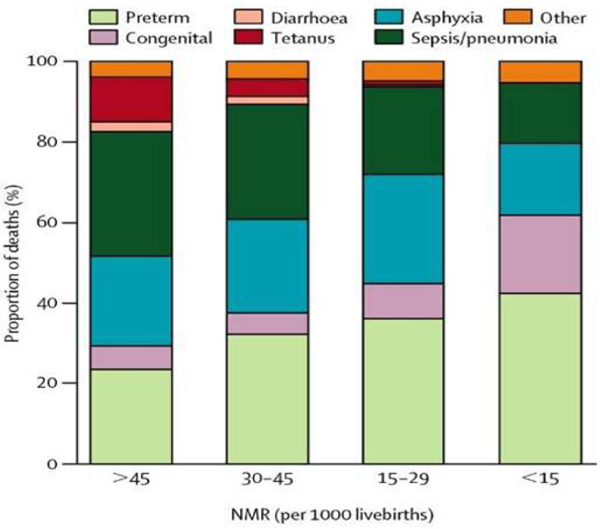
**Percent distribution of neonatal causes of death by level of neonatal mortality showing the increasing proportion of neonatal deaths attributed to preterm birth with lower neonatal mortality rate.** Source: Reprinted from The Lancet, 365, Lawn JE, Cousens S, Zupan J, 4 million neonatal deaths: When? Where? Why?, 10, 2005, with permission from Elsevier [2].

As shown in Figure 3, the proportion of neonatal deaths attributed to preterm births is inversely related to the rates of neonatal mortality, because in countries with very high neonatal mortality, more deaths occur due to infections such as syphilis or tetanus, as well as to intrapartum-related "birth asphyxia" [2]. However, although the proportion of deaths due to preterm birth is lower in LMICs than in HICs, the cause-specific rates are much higher in LMICs than in HICs. For example, in Nigeria the estimated cause-specific rate for neonatal deaths directly due to preterm birth is 13.5 per 1000 compared to the UK where it is under 2 per 1000. This is due to the lack of even simple care for preterm babies. Neonatal mortality rates are higher in LMICs than in HICs, partly because of poorer access to health services and quality of maternal and newborn interventions [5].

Mortality rates increase proportionally with decreasing gestational age (and hence decreasing birth weight). Mortality and morbidity are highest among infants born at less than 32 weeks gestation. Infants born from 32 to 36 weeks represent about 75% of all preterm births and the group of infants who make up the fastest-growing proportion of the preterm births in HICs, with a 25% increase during 1990-2005 [6]. While improvements in medical care have led to improved survival and long-term outcomes among moderately and extremely preterm babies in HICs, these babies still account for the majority of deaths, especially in LICs where even simple care is lacking.

In Southern Brazil, preterm babies experience high mortality rates due to respiratory infections, diarrhea, and other infections that were eight, five, and six times higher, respectively, than rates of term babies [42]. In the United States during 1995-2002, the mortality rate for term newborns was 2.4-3.0 per 1,000 live births. Among babies who were born between 34 and 36 weeks gestation, the mortality rate was 7.9-9.5 per 1,000 live births [43]. Few studies in the literature evaluate gestational age-specific neonatal mortality rates. The comparison of three such studies in Table 4 illustrates the differences in survival among low-, middle-, and high-income countries.

**Table 4:** Gestational age-specific neonatal mortality rates by 1,000 live births for preterm babies

Gestational Age (weeks)	Ilesa, Nigeria, 1996-2000	Pelotas, Brazil, 2004	Scotland, 1985-1994
34-36	48	15	11
32-33	156	61	33
<32	587	370	194
All preterm (<37)	179	66	41

Source: Ilesa, Nigeria (1996-2000) [100], Pelotas, Brazil (2004) (Barros, personal communication 2009), and Scotland (1985-1994) [101]

The major focus in HICs is now on the extremes of gestational age and survival. In a comparative analysis of data from France and England in 1997, 19% and 27% of babies born at less than 26 weeks survived to discharge; 57% and 68% of those born at 26-28 weeks gestation survived to discharge; and 86% and 92% of those born at 28-32 weeks survived to discharge, respectively [44]. In a cohort of extremely preterm infants from the United Kingdom from 1995, 26% of babies born at 24 weeks survived to discharge, and among those born at 25 weeks, 44% survived to discharge [45]. Similarly, in a Canadian cohort of babies born between 1996 and 1997, 57% of babies born at 24 weeks and 76% of babies born at 25 weeks survived to discharge [46].

### Preterm morbidity and long-term sequelae

The complications of preterm birth arise from immature organ systems that are not yet prepared to support life in the extrauterine environment. The response of the infant's organ systems to the demands of the extrauterine environment and the life support provided have an important impact on the infant's short- and long-term health and neurodevelopmental outcomes. These outcomes are also influenced by the etiology of the preterm birth; maternal and family risk factors; and the extrauterine environment, including the neonatal intensive care unit; and the home and community.

Babies born preterm have an increased risk of morbidity due to different mechanisms. Some are directly related to their immaturity, as with hyaline membrane disease due to the lack of pulmonary surfactant, and retinopathy of prematurity due to the excessive use of oxygen to treat hyaline membrane disease. Preterm birth may also be a marker for other problems that produce disease, such as fetal infection and systemic inflammation, which are themselves associated with intracranial haemorrhage, cerebral white matter damage, cerebral palsy, and chronic lung disease (bronchopulmonary dysplasia) [47].

## Stillbirth burden

### Defining stillbirth

The International Classification of Diseases, 10^th^ revision (ICD-10) [48] defines a fetal death as "death prior to the complete expulsion or extraction from its mother of a product of conception, irrespective of the duration of pregnancy; the death is indicated by the fact that after such separation the fetus does not breathe or show any other evidence of life, such as beating of the heart, pulsation of the umbilical cord, or definite movement of voluntary muscles" without specification of the duration of pregnancy. Although birth weight has been the preferred criterion in the ICD to identify a late fetal death, gestational age is an additional requirement for reporting for international comparative purposes. ICD classifies late fetal deaths (greater than 1000 gms or after 28 weeks) and early fetal deaths (500 to 1000 gms or 22-28 weeks) (Table 5).

**Table 5:** GAPPS quality assessment of epidemiological parameters in global estimates using adapted version of GRADE: STILLBIRTH

Epidemiological Parameters	Stillbirth Rate Estimates 2000 (SNL/immpact)	Stillbirth Rate Estimates 2000 (WHO)	Stillbirth Rate Estimates 2005	Intrapartum Stillbirth Rate 2000	Stillbirth Cause-of-death 2005
Defi nition	For international comparison stillbirth rates refer only to late fetal deaths (>1000g or >28 weeks gest). ICD-10 defines a fetal death as «death prior to the complete expulsion or extraction from its mother of a product of conception, irrespective of the duration of pregnancy; the death is indicated by the fact that after such separation the fetus does not breathe or show any other evidence of life, such as beating of the heart, pulsation of the umbilical cord, or definite movement of voluntary muscles» without specification of the duration of pregnancy. The denominator is all live births plus late fetal deaths.			Stillbirths in the last trimester (late fetal deaths) occurring during the time of labour, but excluding major congenital abnormalities. In verbal autopsy data «fresh stillbirth» is used as a surrogate marker for intrapartum stillbirth. The denominator is all live births plus late fetal deaths.	Multiple classification systems are in use. A comparable system to map the results of verbal autopsy data on cause-of-death with more complex classification systems is urgently needed.
Systematic global estimates available? Source and date	Stanton, Lawn et al, Lancet 2006	WHO MPS, 2006	GAPPS, GBD, CHERG, WHO (Stanton, Lawn et al in process)	Lawn, Shibuya, Stein WHO Bull, 2005	GAPPS, GBD, CHERG, WHO (Stanton, Lawn et al in process)
N of countries with VR data used (note if used as reported, new analysis or adjusted)	44 countries with adult VR coverage > 90%; rates adjusted by model coefficient for VR to allow for under reporting	102 countries with SBR data used as reported;	33 VR (plus 11 from EuroPeristat, some of which are VR-based)	Not in Vital registration	Not in vital registration
N of countries with survey-based estimate used (note if used as is, new analysis or adjusted)	1 country, adjusted by model coefficient	102 countries with SBR data used as reported;	In process -	Not in current national surveys	Only 1 national surveys with SB COD data
N of countries where model-based estimate is used (basis of model)	Model-based estimates are used for 128 countries; based on a random eff ects model	88 countries - based on average SBR:ENMR ratio for that region	(in process)	52 countries with data, 141 countries with estimate based unadjusted reported data by country or if no country data on median for WHO subregion (14 subregions)	(in process)
Types of data inputs used in the model	VR, published studies from community and facility-based studies, household surveys, unpublished datasets	Historical data from 12 High Income Countries were used to calculate a ratio of SBR:ENMR, and was applied to generate SBR from ENNMR for high mortality settings	VR, published data from community and facility-based studies, household surveys, unpublished datasets	National registries, published data from community and facility-based studies, unpublished datasets	National registries, published studies from community and facilities, unpublished datasets
N of observations included in estimation dataset	323 observations resulting from searches of 33,714 citations, plus household surveys and additional unpublished datasets	102 country estimates, plus historical trend data from 12 developed countries	437 observations	73 observations resulting from searches of 13,496 citations	~70 datasets
Median year of input data	Median year for High Income Countries = 1998; Median year for Low Income Countries = 1990	Acceptable date range not specifi ed	2000	1995	2000 (range 1981-2008)
Variability in outcome measurement methods	Yes	Yes	Yes	Yes	Yes, includes clinical assessment; medical records, ICD codes, Verbal Autopsy results
Limitations re: population representativeness	Yes; ~20% of observations from sub-Saharan Africa and South Asia rely on data from hospital studies (likely biased)	No, all observations used were national in scope	Yes; ~41% of observations from both sub-Saharan Africa and S/ SE Asia rely on data from hospital studies (likely biased)	Study based data often from non representative populations	Yes, ~68% of low income country obs from hospital data
Generalizability to population of interest (ie., match between burden of disease and geographic distribution of data )	73% of observations in dataset from Low Income Countries; 24% from sub-Saharan Africa; 21% from SE and S Asia	47 Low Income Countries had estimates based on the 1.2 SBR:ENMR ratio from historical High Income Countries	56% of observations from high income countries	Important gaps in the input data, especially: China, central Africa and central Asia	~40% of observations from low income countries
Is there systematic equity assessment	No	No	No	No	No
Global estimate	SBRate = 24 per 1000 births N of stillbirths = 3.2 million	SBRate =24 per 1000 births N of stillbirths = 3.3 million	(in process)	1.02 million	(in process)
Range	Range of SBRates: 19 – 30 per 1000 Range of N’s of stillbirths: 2.5 - 4.1 million	Wide uncertainty assumed, but not quantified	(in process)	0.66 million - 1.48 million	(in process)
Consistency between estimates if more than one series	Good consistency at global, reasonable at regional but poor at country level	to compare when done	none to compare with	none to compare with	
Overall summary of quality of data input	Moderate for High Income Countries, low for Low Income Countries	Moderate/low for High Income Countries, very low for Low Income Countries		Moderate/low for High Income Countries, very low for Low Income Countries	
Overall summary of estimates Quality according to standards for global estimates	Moderate - transparent methods but limited by input data and by adjustments made to 18 countries which increases global total of stillbirths by approximately 1 million	Moderate to Poor - limited by input data, not fully transparent inputs, the output for high mortality settings is dependent on ENMR (some of which are model-based) and multiplying all the ENMRs by 1.2, which increases the global total of stillbirths by approximately 1 million ;		Moderate - limited by input data and median-based method which may be less sensitive than results from a regression-based model	
Priority areas to improve measurement now	1. Increase consistency in use of definitions (weight/gest age cut-off s) 2. Increase quantity and quality of SBR data in vital registration and national audit data 3. Increase quantity and quality of SBR data from household surveys 4. Increase dissemination of country level best estimates of SBRs			1. Agree on a simple, consistent classifi cation system 2. Increase quantity and quality of stillbirth time and cause-of-death data in vital registration and national audit data 3. Increase quantity and quality of SBR data from verbal autopsies 4. Increase dissemination of country level best estimates of SB time and cause-of-death	

It should be noted that "stillbirth" is not a technical term. In this article "stillbirth" refers to late fetal deaths to conform to the WHO recommendation that late fetal deaths be reported for purposes of international comparison. The rationale for restricting international reporting to stillbirths of greater than 1000 gms or after 28 weeks is to assure comparability, as the countries where most stillbirths occur mostly still do not capture even these larger more mature deaths reliably and data remain uncertain [49]. In countries lacking neonatal intensive care, few babies below the gestational age of 30 weeks survive [31]. However, in many countries where neonatal intensive care units are available, the gestational age for viability has decreased, and the gestational age criterion to define stillbirth has been adapted accordingly. Current gestational age thresholds for stillbirth vary from 16 to 28 weeks of gestation across countries.

### Stillbirth rates estimates

Prior to 2006, no organization had published global, regional or country-specific stillbirth rates. Two global series of stillbirth estimates for the year 2000 were published in 2006 (hereafter referred to as the SNL/immpact and WHO estimates) [7,50], with both exercises generating estimates of just over three million stillbirths (3.2 million, with wide uncertainty: 2.5-4.1 million; and 3.3 million, respectively). SNL/immpact represents a collaboration between Saving Newborn Lives/Save the Children USA and the Initiative for Maternal Mortality Programme Assessment, at the University of Aberdeen, Scotland.

**Figure 4:**
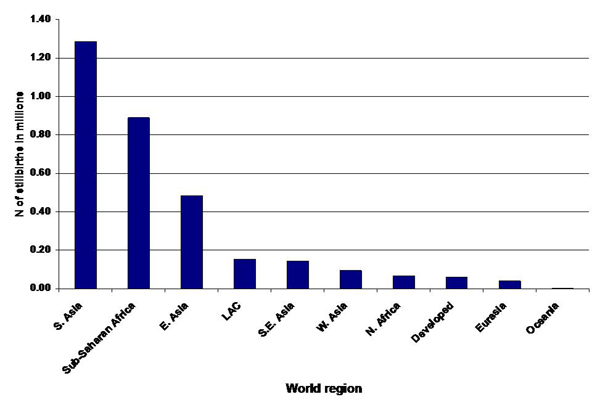
**Estimated global number of stillbirths by world region, 2000.** Source: Reprinted from The Lancet, 367, Stanton C, Lawn JE, Rahman H, Wilczynska-Ketende, K, Hill K, Stillbirth rates: delivering estimates in 190 countries, 8, 2006, with permission from Elsevier [7].

Figure 4 presents the SNL/immpact numbers of stillbirth by region.

Given the very different methods used in these two estimation exercises and the dearth of stillbirth data available from developing countries, the results are remarkably similar. Table 6 summarizes regional stillbirth rates from the two series of estimates. Stillbirth rates are very similar for sub-Saharan Africa and South Asia (32 per 1000 births). However, there is little agreement between the remaining regional estimates and even less agreement at the country level, where the data are most needed for planning purposes; for example, two- to three-fold differences in both directions exist between the two series of estimates for some countries (data not shown). Figure 4 presents the estimated number of stillbirths by world region.

**Table 6:** Comparison of stillbirth rate estimates at regional levels

	Stillbirth Rate per 1,000 births	
	
World Region (WHO regions)	WHO estimate	SNL/immpact estimate (95% CI)
World	24	23.9 (18.8-30.5)
HICs	4	5.3 ( 4.2- 6.8)
LMICs	26	25.5 (20.0- 32.5)
North Africa	16	18.6 (14.1-24.7)
Sub-Saharan Africa	34	32.2 (25.4-40.9)
Latin America/Caribbean	10	13.2 (10.4-16.7)
East Asia	19	23.2 (18.3-29.5)
South Asia	34	31.9 (25.0-40.7)
Southeast Asia	18	12.7 (10.0-16.0)
West Asia	16	18.9 (14.3-24.9)
Eurasia	23	12.2 ( 9.5-15.5)
Oceania	17	15.8 (12.4- 20.1)

Sources: [7,50]

The methods for both series of stillbirth estimates have been summarized in Table 5. Any global estimation exercise is by definition an attempt to make the best of sub-optimal data. Both series of estimates suffer from a lack of quantity and of quality input data. This leads to decisions in the modelling process that are easy targets for criticism. Our summary assessment of these two exercises is "moderate" at best when judged according to the criteria outlined in Table 5. An updated series of stillbirth rates and numbers for 2005 will be undertaken jointly by WHO and CHERG with GAPPS and undergo external review prior to the next global burden of disease exercise.

### Availability of stillbirth rate data

In HICs, national vital registration systems usually have high coverage and reasonably reliable cause-of-death data for live births, but the stillbirth data are often more questionable [7]. Globally, only about 2% of late stillbirths are accounted for via vital registration. In countries lacking complete vital registration on stillbirths, but with high institutional birth rates, health facility-based data are also an important source of representative data on pregnancy outcomes. In LMICs, by far the largest source of data on stillbirths comes from population-based household surveys. Other sources include demographic surveillance sites, or special studies. In LMICs lacking high institutional birth rates, health facility data can still be a valuable resource if compiled regionally or nationally, especially if selection bias is taken into account. Notable examples are the Latin American Center for Perinatology (CLAP) database [51] and the South African Perinatal Identification Programme [52,53].

### Stillbirth causes of death

Currently there are no global, systematic estimates for stillbirth causes of death. Where data do exist, the lack of comparability across studies greatly inhibits interpretation. More than 30 different stillbirth classification systems have been identified in the literature [54], with some encompassing up to 37 causes [55]. Most focus on stillbirths in HICs where determination of the most prevalent causes requires fetal surveillance and sophisticated diagnostics [49]. Later in the article, options for improving stillbirth cause-of-death comparability will be discussed.

**Figure 5:**
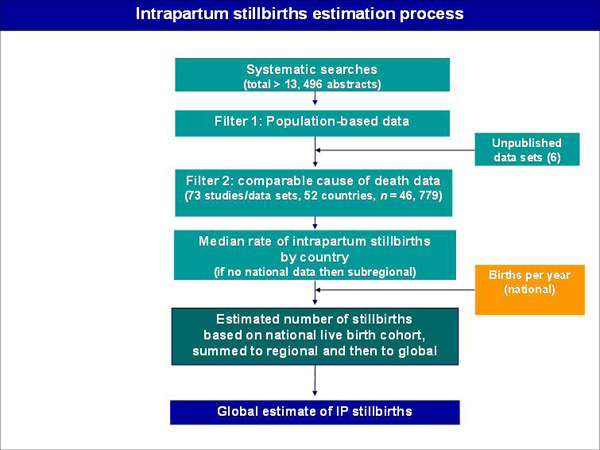
**Data sources and methods for estimates of intrapartum stillbirth rates for 192 countries.** Source: Reprinted from Bulleting of the World Health Organization, Lawn JE, Shibuya K, Stein C, No cry at birth: global estimates of intrapartum stillbirths and intrapartum-related neonatal deaths, 2005, with permission from WHO Press [56].

Comparable data regarding the timing of stillbirths relative to delivery are more widely available. Intrapartum stillbirths are generally defined as stillbirths occurring after the onset of labour, or as "fresh stillbirths" (with skin still intact, implying death occurred less than 12 hours before delivery) weighing more than 1,000 grams and more than 28 weeks of gestation, but exclude severe lethal congenital abnormalities [56]. This increased availability of data permitted publication in 2005 of intrapartum stillbirth rates for 192 countries. Details regarding the input data for this series of estimates are included in Figure 5 and methods are summarized in Table 5.

Based on these estimates, one million intrapartum stillbirths occur annually (uncertainty bounds: 0.66-1.48), representing one-third of stillbirths globally [56]. Despite the caveats inherent in the interpretation of the intrapartum stillbirth estimates, these estimates clearly highlight the magnitude of loss of life just minutes and hours prior to birth. Hospital-based studies suggest that from 25-62% of intrapartum stillbirths are avoidable with improved obstetric care and more rapid responses to intrapartum complications, including reducing delays in seeking care from home [57-60].

At the time of writing of this supplement, a systematic review of the literature on stillbirth cause-of-death is underway. Data permitting, the distribution of causes of stillbirth will be estimated, using methods similar to those used to estimate neonatal cause-of-death [61]. Approximately 65 studies from 36 countries have been identified that provide at least minimal stillbirth cause-of-death data. Data have been extracted into the following categories: congenital abnormality (physically visible); maternal conditions (including pregnancy-induced hypertension, eclampsia/preeclampsia, diabetes, other antenatal); antepartum hemorrhage (abruption); infections (including syphilis, other maternal and other fetal infection); intrapartum stillbirth (including obstruction, and breech); preterm labor of undetermined cause; other and unclassifiable.

## Opportunities to improve data on preterm births and stillbirths

### Preterm birth data improvement

#### Improving measurement of preterm birth prevalence

There are many opportunities to improve data now in both low- and high-income countries (Table 7). The definition for preterm birth (less than 37 weeks of completed gestation) is well-known. The challenge is the current low priority given to collecting gestational age data, and the complexity of measurement (apart from the use of last menstrual period). Further effort is needed to influence the content of midwifery and medical pre- and in-service education and to establish gestational age assessment as an integral component of routine care.

**Table 7:** Improving country level data for preterm birth – what can be done now and what are the research priorities?

	Opportunities Immediately Available		
		
Opportunities	High-Income Settings	Low-Income Settings	Research Priorities (Focus on high mortality, low quality data settings)
Comparable case definitions and better definitions of phenotypes	Use 37 completed weeks of gestation but also advance data for very preterm (<34 weeks) and moderate (34-36.9) as well as for spontaneous and medically induced preterm birth	Prioritize improved collection of representative population-based data preterm prevalence as a key starting point	Development of simple and feasible proxy indicators for gestational age (e.g., weight)
Mechanisms for data collection	Include gestational age and birth weight data on birth certificates and perinatal death certificates. Cross-link data from vital registration and health facility surveillance.	Improve vital registration systems. Use specific death certificates for stillbirths/neonatal deaths and include gestational age and birth weight data on birth certificates	Validation of approaches to assess gestational age through household survey data
Cause-of-death attribution mechanisms	Use vital registration specific death certificates for stillbirth and neonatal deaths. Revise current ICD codes for preterm birth to reflect change in focus from birth weight to gestational age	In large-scale surveys, follow-up interviews with a verbal autopsy for recent stillbirth and neonatal deaths. Use standardized verbal autopsy tool, case definitions and hierarchical attribution for cause-of-death. Provide clear guidelines for when to attribute death to preterm complications.	Evaluation of the use and reliability of a standardized verbal autopsy tool, case definitions and hierarchy of causes of death. Development of verbal autopsy classification software which provides greater consistency and costs less than expert assessment of verbal autopsy data
Counting avoidable factors, using data in programmes	Increase the number of national audit systems Consider confidential enquiry for neonatal deaths and stillbirths, as well as maternal deaths	Develop or modify audit systems linking maternal/fetal and neonatal deaths. Compile national data and/or promote sentinel sites in varying health system contexts to ensure that the information is useful for policy prioritization, even if not representative of the population. Consider focusing on few indicators initially (e.g. Intrapartum Case Fatality Rate). Use existing data (e.g., facility birth registers) for local monitoring and programmatic decision-making.	Evaluation of simple audit tools and a mechanism to maximize resultant change in policy and programs.

In HICs, gestational age assessment has surpassed birth weight as the measurement of choice, with a much closer correlation to short- and long-term outcomes. A number of methods exist for the assessment of gestational age. In middle-income countries, gestational age is increasingly available, even with ultrasound dating (the gold standard). In most countries, a very small proportion of births have reliable gestational age assessment. Even estimates of gestational age based on last menstrual period are often not recorded or known, particularly in African settings. In most survey-based data, women are asked to state their gestational age in completed months. This is the practice in the DHS contraceptive calendar, for example.

##### Option 1: Birth weight as a surrogate measure

In LMICs, low birth weight is often used as the criterion for identifying preterm births given the paucity and quality of self-reported data on gestational age. Reliance on LBW is problematic, however, as 58% of babies in LMICs are not weighed at birth (Table 8), and home- based births, those most likely to be of low birth weight, are not represented [23]. In middle-income countries, notably in Latin America, many countries have a record of birth weight for the majority of babies (83%), but in South Asia and sub-Saharan Africa, where the majority of neonatal deaths occur, only a fourth to a third of babies have a record of birth weight. These figures parallel the coverage of skilled attendance at birth, though even with a facility-based birth by a skilled attendant, the birth weight may not be recorded due to a lack of scales, skilled staff, and standard protocols [13].

**Table 8:** Percent of live births that are not weighed by world region

World Region (UNICEF)	Percent of Births NOT Weighed at Birth
South Asia	74
Sub-Saharan Africa	65
Middle East and North Africa	60
East Asia and Pacific	30
CEE/CIS	21
Latin America and Caribbean	17

Source: Data from Blanc AK, Wardlaw T, 2005 [23]

Table 9 shows the proportion of preterm babies in different birth weight groups. Only about half of the newborns at 2000-2499-grams were born preterm. These data suggest that using a cut-off of 2000 grams may be more appropriate than the traditional LBW definition in identifying preterm births. The two studies described in Table 9 are from Latin America, and these proportions may differ in other regions, such as South Asia where intrauterine growth restriction is highly prevalent. When data are available for birth weight and age-at-death of stillbirths and neonatal deaths, a simple cross tabulation of birth weight by age-at-death can be a useful guide for programmatic priority setting [62]. For example, full-size babies dying during birth have very different solutions to very small babies dying after birth.

**Table 9:** Distribution of preterm births according to birthweight group. Uruguay

Birth Weight (Grams)	Uruguay 1986-2003 (n=476,571)	Pelotas 1982, 1993, 2004 (n=14,117)
3,000+	3.0%	3.4%
2,500-2,999	14.6%	13.4%
2,000-2,499	49.0%	45.0%
1,500-1,999	84.8%	88.7%
<1,500	93.4%	97.5%
All	10.7%	11.0%

Source: PAHO/WHO Latin American Center for Perinatal Health (Barros, unpublished permission granted by author)

##### Option 2: Clinical assessment of gestational age

Given the need for a paradigm shift to use gestational age instead of birth weight for the identification of preterm births, the possibility of simplified gestational age assessment by lower cadres of workers is of interest. A recent systematic review of methods for gestational age assessment identified 17 different methods using a combination of neurological and physical criteria or physical criteria alone [63]. Methods requiring complex technology or neurological assessment alone were excluded. Of these 17 methods, five were considered "complex," nine were "intermediate," and three were "simple," based on the number of characteristics examined.

As compared against varying standards (only some of which were ultrasound) all methods were accurate within plus or minus three weeks. The number of methods to choose from and the varying levels of complexity allow for recommendations to be made appropriate to two settings: tertiary care hospitals and district-level health facilities. Further uptake of these methods are needed by international and medical professional associations to influence the content of midwifery and medical pre- and in-service education as a means of establishing gestational age assessment as an integral component of routine care. Evaluation of use at large-scale settings and data validity could further refine recommendations by setting. However, none of the methods which were compared against an acceptable standard were applied by community health workers. Hence, further research is required to identify the most feasible and acceptably accurate methods for community-based gestational age assessment.

#### Improving measurement of other parameters related to the burden of preterm birth

A new analysis would be required to better delineate the effect of preterm compared to term gestational age, to define the risk of varying gestational ages for death, and to separate direct from indirect risks. Individual-level data on birth weight, gestational age, mortality outcome and ideally comparable cause-specific mortality would be required for such an analysis (Table 1).

To improve the assessment of long-term outcomes of preterm birth, particularly impairment outcomes, an international consensus group is required to agree to standard definitions for these parameters. Protocols and tools are required to ensure standard measurement, especially for disability and cognitive function at various ages.

### Stillbirth data improvement

#### Improving the data on stillbirth rates and numbers

Table 10 summarizes a number of opportunities that are immediately available to improve stillbirth data through existing data collection mechanisms.

**Table 10:** Improving country level data for stillbirths – what can be done now and what are the research priorities?

	Opportunities Immediately Available		
		
Opportunities	High-Income Settings	Low-Income Settings	Research Priorities (Focus on high mortality, low quality data settings)
Comparable case definitions for stillbirth	Use 28 week cut-off for international comparisons and 22 week cut-off for High-Income Country comparisons. Local definitions can be used for local purposes.	Prioritize improved collection of representative population-based data for last trimester and intrapartum stillbirths.	Development of simple and feasible proxy indicators for gestational age (e.g., weight)
Mechanisms for counting all births, (including stillbirths)	Improve vital registration data by establishing specific death certificates for stillbirth and neonatal deaths. Cross-link data from vital registration and health facility surveillance.	Increase attention to training and field supervision for DHS-type household surveys which rely on retrospective reporting of all births. Consider adding stillbirth data collection to MICS surveys. Analyze existing pregnancy loss data from sentinel surveillance sites and increase the number of sentinel surveillance sites which prospectively collect stillbirth data. Improve vital registration systems and register stillbirths. Use specific death certificates for stillbirths/neonatal deaths.	Validation of existing approaches for pregnancy loss data collection compared to pregnancy loss data from sentinel surveillance sites
Classification for stillbirth cause-of-death	Obtain consensus on a single classification system with a limited number of programmatically relevant, comparable categories, that can be distinguished in low income settings through verbal autopsy, but can also be directly incorporated into more detailed sub groups necessary in high income settings		Evaluation of validity and feasibility of a simple standard classification system for stillbirth cause-of-death
Cause-of-death attribution mechanisms	Use vital registration specific death certificates for stillbirth and neonatal deaths. Revise current ICD codes for stillbirths to reflect changes in attribution of cause-of-death since the 1980s.	In large-scale surveys, follow-up interviews with a verbal autopsy for recent stillbirth and neonatal deaths. Use standardized verbal autopsy tool, case definitions and hierarchical attribution for cause-of-death.	Evaluation of the use and reliability of a standardized verbal autopsy tool, case definitions and hierarchy of causes of death. Development of verbal autopsy classification software which provides greater consistency and costs less than expert assessment of verbal autopsy data
Counting avoidable factors, using data in programmes	Increase the number of national audit systems .Consider confidential enquiry.	Develop or modify audit systems linking maternal/fetal and neonatal deaths. Compile national data and/or promote sentinel sites in varying health system contexts to ensure that the information is useful for policy prioritization, even if not representative of the population. Consider focusing on few indicators initially (e.g. Intrapartum Case Fatality Rate). Use existing data (e.g., facility birth registers) for local monitoring and programmatic decision-making.	Evaluation of simple audit tools and a mechanism to maximize resultant change in policy and programs.

Abbreviations: Demographic Health Surveys (DHS), Multiple Indicator Cluster Surveys (MICS)

##### Option 1 - Vital registration

Improved measurement of stillbirths in HICs requires a focus on highly standardized reporting of stillbirths via vital registration or other comprehensive national registries. The most important data intervention is the establishment of a stillbirth death certificate. Given the plethora of data available from HIC health facilities, standardized reporting is entirely feasible. At issue is the political will to demand such an intervention. Establishment of a stillbirth death certificate could address both improved counting of events, as well as improved standardization of the causes of stillbirth.

In LMICs, one should capitalize on the current increased interest in improving vital registration by also introducing a standard perinatal death certificate. Complete registration may be a distant goal, but as the foundation for improved data is being established, stillbirths should be included or countries will miss the opportunity to show mortality change concurrent with the implementation of maternal and neonatal programs.

At the international level, seizing the opportunity of the upcoming revision of ICD codes to reflect recent advances in diagnosing stillbirth cause-of-death is essential to future data improvement. Regarding the identification of avoidable factors for the prevention of stillbirth, expanding the use of national audits or other forms of confidential inquiry is recommended. In addition to investigating traditional deficiencies in quality of care, these audits can be adapted to specific contexts to also examine socioeconomic disparities and demographic or behavioral characteristics of the population of interest.

##### Option 2 - Population-based surveys

The Demographic and Health Survey (DHS) website has posted national data on stillbirth rates for 49 surveys from 38 countries [64]. These surveys are by far the largest source of national data from LMICs. Given the lack of vital registration data on stillbirths in LMICs, reliance on survey-based estimates is inevitable for the near future, and given that 98% of stillbirths occur in LMICs—this data source cannot be ignored. The majority of DHSs consist of a complete live birth history for each woman of reproductive age in the sample. Many also include a contraceptive calendar in which monthly data on each respondent's contraceptive use, pregnancy status, and pregnancy outcomes are collected for the 60-month period prior to interview. These data permit calculation of stillbirth rates.

DHS stillbirth rates range from 3.4 per 1000 (in Ukraine) to 37.0 per 1,000 (in Bangladesh). Excluding the surveys in Bangladesh and Nepal, DHS stillbirth rates do not surpass 20 per 1,000. However, the Bangladesh DHS estimate is similar to the high-quality estimate from demographic surveillance data in Matlab, Bangladesh [65].

Evidence from countries with adequate historical data suggest that an SBR:ENMR ratio of approximately 1.2 can be expected in high mortality countries [50]. Only 5 of the 49 DHS surveys show ratios greater than one. For sub-Saharan African countries, the regional (population- based) ratio is only 0.55, and ranges from 0.61-0.64 for the remaining regions, suggesting extreme under-reporting in the large majority of these countries. Moldova stands out as an extreme outlier with a ratio of 3.2, suggesting likely misclassification between stillbirths and early neonatal deaths. In a separate analysis of stillbirth rates from multiple data sources, DHS calendar-based stillbirth estimates were found to be approximately 30% lower than other population-based studies, after controlling for other study and population characteristics [7]. As currently implemented, the contraceptive calendar is not a reliable source of stillbirth data.

Over the past 20 years limited research attention has been applied as to how best to collect population-based pregnancy loss data. In 1989, Casterline analyzed the pregnancy loss data in 41 World Fertility Surveys and concluded that these pregnancy histories in their various formats detected from 50-85% of recognizable pregnancy losses, as compared to results from prospective, clinical studies in Western countries [66]. As expected, late fetal losses tended to be better reported than earlier miscarriages. Garenne noted the highly reliable reporting on perinatal mortality in Niakhar, Senegal, when comparing pregnancy history data to DSS data [67]. Goldman et al. [68], Westoff et al. [69], and Becker and Sosa [70] studied the effects of using a truncated pregnancy history in Peru, the Dominican Republic, and Costa Rica, with varying results depending on the outcome studied. Stanton found the reliability of reporting stillbirths in two national DHS-type surveys using pregnancy histories from the Philippines to be lower than for early neonatal or infant deaths [71].

To date the most rigorous examination of the validity of self-reported pregnancy outcome data was undertaken by Espeut in Bangladesh: comparing DSS data from Matlab, Bangladesh, matched to respondents in a DHS survey in which respondents were randomly assigned a questionnaire with a live birth or pregnancy history. In summary, a 91% sensitivity rate was found for reporting in the survey on stillbirths. In contrast, the sensitivity rate for early neonatal deaths varied from 79-81% in live birth and pregnancy histories; among stillbirths in the DSS, 3% were misclassified as live births, and 9% were misclassified as abortions (suggesting difficulty in recalling gestational age) compared to self-report in the surveys [72]. The goal of future validation efforts should not be restricted to identification of the highest quality approaches but should quantify the loss of data quality in choosing, for example, a truncated vs. complete live birth or pregnancy history, or a survey covering wide-ranging issues vs. a highly focused questionnaire. Immediate progress can be made by carefully reviewing the wide variation in current data collection processes—including the formulation of questions that would elicit reporting on pregnancy losses. Likewise, assuring increased attention to the definition of stillbirth during interviewer training and improving supervision in the field could also lead to immediate improvements in data quality.

##### Option 3 - Demographic surveillance sites and special research studies

Demographic surveillance sites (DSS), in which the vital events and background characteristics associated with all residents are recorded prospectively, should be an important data source on early pregnancy loss, stillbirths, and preterm births. INDEPTH, a network of researchers from DSS around the world, promotes the registration of pregnancy and pregnancy outcomes as a means of early registration of births but also for identifying stillbirths and abortions [73]. Although the collection of pregnancy loss data is highly recommended in DSS, it is unclear how many current DSS actually collect pregnancy loss data, and among those that do, how many regularly or ever report such results. Few DSS data could be located in the published or web-based literature. In contrast, the DSS in Matlab, Bangladesh, includes stillbirth data in their routine reporting [65]. The evaluation of existing but publicly unavailable data on late pregnancy loss from demographic surveillance sites demands immediate attention and could potentially offer important clues to improved data collection.

#### Improving stillbirth cause-of-death data

While lack of data on stillbirth cause-of-death is a large hurdle to overcome, another major barrier is the lack of a classification system that is feasible for low-income countries and which is based on categories that can be mapped alongside more complex classifications which are useful in high-income settings [49]. Currently, two- thirds of the world's stillbirths lack programmatically meaningful cause-of-death categories which could be used to inform prevention strategies.

Stillbirth classification systems have proliferated over the years and a review suggests at least 33 are in use [54]. Most of these are designed for high-income countries and involve laboratory and pathological examination of the baby and the placenta, so are impractical for use when the only information for most stillbirths is through verbal autopsy occurring a year or even longer after the loss. International consensus for standard classification and comparable attribution of cause are essential to improve the comparability and use of stillbirth cause-of- death data. This can only be achieved if the classification system is practically applicable and serves the needs of high- as well as low-mortality settings.

High-mortality settings require broad causal categories which can be distinguished through simple clinical observations or even through verbal autopsy and which are programmatically relevant in that they identify conditions associated with large numbers of deaths. One useful distinction for stillbirth prevention strategies is between macerated (antepartum) and fresh (intrapartum) stillbirths. Rates of fresh stillbirths are assumed to reflect the quality of intrapartum care (care in labor), while rates of macerated stillbirths are assumed to reflect the quality of fetal growth and of care during the antenatal period. The antepartum/intrapartum distinction can generally be explored in verbal autopsy studies with questions pertaini ng to the appearance of the infant's skin. Such questions have been used and are believed to be well understood by respondents, though they have not yet been systematically validated. There is some potential for misclassification between these categories. For example, in settings with major delays in access to health care, stillbirths may die during labor, but not be delivered for days by which time they are classified as macerated. Conversely, some intrapartum stillbirths may be due to infections or congenital causes. Also, women who have delivered stillbirths may not be shown the infant, and therefore could not adequately respond to questions about the infant's appearance. The extent of this misclassification may vary locally and requires further research [56].

**Figure 6:**
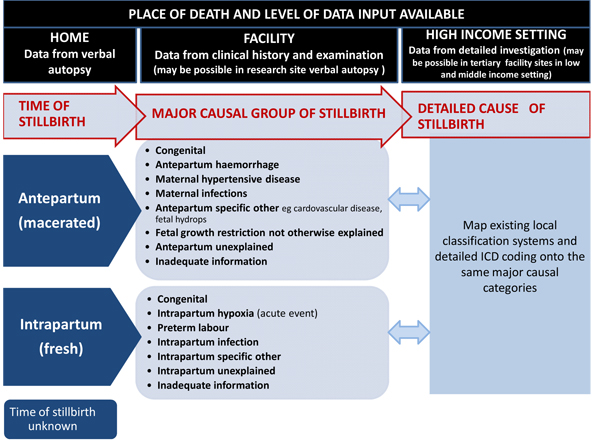
**Classifi cation system for stillbirth cause-of-death.** Source: Reprinted from BMC Pregnancy & Childbirth, Lawn JE, Yakoob YM, Haws, RA, Soomro T, Darmstadt GL, Bhutta ZA, 3.2 million stillbirths: epidemiology and overview of the evidence review, 2009, with permission from BMC [49]

Once these two major categories (antepartum and intrapartum) are defined, a more detailed set of programmatically relevant causal groups can be distinguished. This intermediate level of detail is possible with clinical data and achievable in most facility deaths in LMICs (e.g., the South African National Saving Babies data) [74,75]. For high-income countries, the existing complex classification systems often require sophisticated investigation but can be mapped onto simpler clinical categories (Figure 6). In verbal autopsy data and even in clinical assessment, some causal groups will be systematically underestimated. For example, congenital abnormalities are underestimated even in high-income countries but are markedly underestimated in verbal autopsy data because only obvious external abnormalities are detected and important internal structural and metabolic disorders are missed. Data from the literature show that around 5-15% of stillbirths are attributed to a congenital cause. Another important cause of stillbirth that is often missed is maternal syphilis. Figure 6 proposes groupings allowing a layered approach with increasing complexity of causal attribution in varying settings. Much could be learned by reclassifying existing data on stillbirth causes of death using the classification system proposed in Figure 6 (or some adaptation thereof) via collaboration with the original authors responsible for data collection. Such an exercise would quickly and inexpensively test this proposed classification and identify any caveats in the interpretation of the results.

Such a classification system for stillbirth cause-of-death would allow comparability between different data collection systems, such as verbal autopsy and more complex data systems (Figure 6). Several verbal autopsy tools now exist, thus gaining consensus on a standardized verbal autopsy tool would be an important advance. Such a tool would need to be tested in a wide variety of contexts. Data on avoidable factors contributing to stillbirths could also be addressed within the verbal autopsy questionnaire by adding a social autopsy module covering questions regarding care-seeking and beneficial or harmful traditional practices, for example. Much more in-depth information would be available through the use of a stillbirth audit, and there are a number of LMICs attempting to increase the coverage and quality of their audit networks. South Africa is an example of a country which has achieved both high coverage and high quality of perinatal audit data that are used for national decisionmaking [76].

## Conclusion

Despite more than three million annual stillbirths and approximately one million neonatal deaths directly due to preterm birth, these burdens and the associated loss to families and nations are rarely highlighted in global health policy and research agendas. The impact of the combined numbers of deaths from stillbirth and preterm birth, plus the morbidity and long-term disability associated with preterm birth, is considerable. Clinical researchers and epidemiologists face formidable barriers in collecting and analyzing data about prevalence and interventions, particularly in South Asia and sub-Saharan Africa where two-thirds of these events occur. The places with the highest risk currently have the least information available. Yet, the quantity and quality of information could be improved, even in the short-term by: (1) seizing opportunities to add or test the measurement of stillbirths and preterm births to ongoing data collection mechanisms; (2) using consistent definitions and classification systems across current data collection mechanisms and research studies; and (3) improving global estimates for both outcomes. Research into etiologic mechanisms responsible for stillbirth and preterm birth has been hampered by the lack of standardized definitions and measurement protocols for assessing these outcomes. The global economic burdens related to these outcomes remain a significant research gap.

From this review, the priority gaps in existing estimates and in the data for effective program design include the following:

• Systematic estimates for causes of stillbirth are required to increase visibility and prioritize action to reduce these deaths. Agreement around a simplified classification system is a key step to underpin global estimates.

• The lack of systematic country-level estimates for the prevalence of preterm birth, based on well-defined and standard phenotype classification, is an important gap affecting the visibility of preterm birth globally. The lack of information for preterm prevalence is most marked in Africa and the Eastern Mediterranean. Virtually no consistent data on preterm prevalence trends are available from LMICs. The development of methods to permit reliable population-based data on trends in preterm birth in these countries is a key priority.

• New analysis is required to better define the risk of death at varying gestational ages, and to separate direct from indirect risks. Input data sets would need to include individual-level data on birth weight, gestational age, mortality outcome, and ideally, comparable causes of death.

• Acute morbidity and long-term sequelae of preterm birth remain virtually unstudied in LMICs, despite the fact that survival is now increasing in some of these settings. Tracking morbidity is crucial. Standard tools and protocols to assess morbidity and long-term sequelae across varying cultures are lacking. Attempts at these global estimates are severely hampered by the lack of data.

Opportunities highlighted by this review that could improve the availability and quality of data, even in the short term, include:

• Improve the capture and quality of pregnancy outcome data through household surveys, which is the main data source for the countries with 75% of the global burden, and undertake validation studies. The expanded number of demographic surveillance sites currently functioning in various LMICs offer excellent opportunities to compare prospective versus retrospective reporting on pregnancy outcomes.

• Increase awareness of, and compliance with, standard definitions for stillbirth and preterm birth, and more frequently include stillbirth and gestational age data in existing data collection systems (vital registration, facility-based data and research studies). Current ICD 10 codes for both stillbirth and preterm birth need to be updated to reflect definitions currently in use and advances in understanding made in the last decade. A simplified classification system for stillbirth cause-of- death could also be incorporated into the ICD 11. This would allow data from a standardized verbal autopsy tool and other data collection systems in LMICs to improve input data for future global estimates.

• Expand and strengthen the coverage and quality of existing data collection mechanisms, especially vital registration, and facility data by instituting a standard death certificate for stillbirth and neonatal death linked to revised International Classification of Diseases coding.

• Validate a simple, standardized classification system for stillbirth cause-of-death that is feasible though verbal autopsy.

• Improve systems and tools to capture acute morbidity and long-term impairment outcomes following preterm birth and other adverse pregnancy or neonatal events.

In addition to these priority actions to improve preterm birth and stillbirth data in the immediate future, there is an extensive research agenda around the epidemiology of preterm births and stillbirths and many possible research questions too detailed to list here. The final article in this report presents a Global Action Agenda developed by global stakeholders at the GAPPS International Conference on Prematurity and Stillbirth held in May 2009, and includes short- and long-term objectives related to the epidemiology of preterm birth and stillbirth [16].

The numbers discussed in this report are large—on par with the issues considered the greatest priorities in global health today, and indeed larger than some that receive major attention, such as two million annual HIV/AIDS deaths. Yet, preterm birth and particularly stillbirth are not included amongst global priorities. This invisibility is partly an issue of data, but remains a reality despite increasing quality and progress for global estimates. Another critical issue is the value put on a baby's life—a newborn baby remains the most vulnerable human and a preterm newborn is even more vulnerable.

Each of these losses is a bereavement for families and may leave a deeper scar than a death which is openly acknowledged and mourned. Long-term follow-up studies show that 20 years after a stillbirth, a woman may remain in a delayed grief response [77,78]. The societies where stillbirth and preterm birth have become priorities are those where such babies are expected to live, and women and families can express their loss. Indeed, the power of these families to use data for change may be likened to the power of individuals who lost loved ones from HIV/AIDS and advocated successfully for change. Data alone will not result in change until society and leaders recognize that these deaths are a loss that can and must count and be prevented.

## Additional File

Additional file 1 shows relevant definitions

## Authors' contributions

The article was written by JEL and CS and reviewed by MG, CR, TN and the GAPPS Review Group.

## Competing interests

The authors declare that they have no competing interests.

## Supplementary Material

Additional file 1 Click here for file
