# Supplementary material for: Global report on preterm birth and stillbirth (1 of 7): definitions, description of the burden and opportunities to improve data
Source: BMC Pregnancy Childbirth. 2010 Feb 23;10(Suppl 1):S1. doi: 10.1186/1471-2393-10-S1-S1 (PMC2841772; doi:10.1186/1471-2393-10-S1-S1)
Supplement: Additional file 1 [file 1471-2393-10-S1-S1-S1.doc]

**Additional File 1**

# Geographic Regions

Throughout this report, when countries are the units of analysis, the World Bank classification for low-, middle-, and high-income countries is used. The review is especially focused on LMICs, and may also be significant for HICs. Although there are more detailed regional classifications, such as the 14 regions used by the Global Burden of Disease, this report usually presents the regional information according to the six World Health Organization (WHO) regions: AFR (Africa), AMR (Americas), EUR (Europe), EMR (Eastern Mediterranean), SEA (South East Asia), and WPR (Western Pacific), as many estimates of mortality use this classification (Bryce et al. 2005). Any depiction of boundaries does not reflect a statement on the legal status of any country.

**List of abbreviations used**

CHERG Child Health Epidemiology Reference Group

CHNRI Child Health and Nutrition Research Initiative

CEmOC Comprehensive Emergency Obstetric Care

CLD Chronic Lung Disease

CNS Central Nervous System

DHS Demographic and Health Survey

DSS Demographic Surveillance Sites

GAA Global Action Agenda

GBD Global Burden of Disease

HICs High-Income Countries

HIV Human Immunodeficiency Virus

ITN Insecticide Treated Nets

IPI Interpregnancy Interval

IPTp Intermittent Presumptive Treatment During Pregnancy (for Malaria)

LBW Low Birth Weight

LMICs Low- and Middle-Income Countries

MDG Millennium Development Goal

MICS Multiple Indicator Cluster Survey

MNCH Maternal, Newborn, and Child Health

NICU Neonatal Intensive Care Unit

PMR Perinatal Mortality Rate

PTB Preterm Birth

RCT Randomized Controlled Trial

ROP Retinopathy of Prematurity

SB Stillbirth

SBR Stillbirth Rate

TBA Traditional Birth Attendant

UNICEF United Nations Children’s Fund

VLBW Very Low Birth Weight

VR Vital Registration

WHO World Health Organization
